# Student Preferences on Gaming Aspects for a Serious Game in Pharmacy Practice Education: A Cross-Sectional Study

**DOI:** 10.2196/mededu.3754

**Published:** 2015-05-11

**Authors:** Huan Ying Chang, David Yan Hong Poh, Li Lian Wong, John Yin Gwee Yap, Kevin Yi-Lwern Yap

**Affiliations:** ^1^ National University of Singapore Department of Pharmacy, Faculty of Science National University of Singapore Singapore Singapore; ^2^ Computer Centre National University of Singapore Singapore Singapore

**Keywords:** gaming aspects, pharmacy-related serious game, pharmacy practice education, reward systems, game settings, storylines, viewing perspectives, gaming styles

## Abstract

**Background:**

Serious games are motivating and provide a safe environment for students to learn from their mistakes without experiencing any negative consequences from their actions. However, little is known about students’ gaming preferences and the types of serious games they like to play for education.

**Objective:**

This study aims to determine the types of gaming aspects that students would like to play in a pharmacy-related serious game.

**Methods:**

A cross-sectional study was conducted using a self-administered survey, which obtained students’ responses on their preferences regarding various gaming aspects (reward systems, game settings, storylines, viewing perspectives, and gaming styles) and for a hypothetical gaming scenario (authentic simulation or post-apocalyptic fantasy). Descriptive statistics, chi-square, and Fisher’s exact tests were used for statistical analyses.

**Results:**

Response rate was 72.7% (497/684 undergraduates). The most popular game reward systems were unlocking mechanisms (112/497, 22.5%) and experience points (90/497, 18.1%). Most students preferred fantasy/medieval/mythic (253/497, 50.9%) and modern (117/497, 23.5%) settings, but lower year undergraduates preferred modern settings less than upper year seniors (47/236, 19.9% vs 70/242, 28.9%, *P*=.022). Almost one-third (147/497, 29.6%) preferred an adventurer storyline or an authentic pharmacy-related plot (119/497, 23.9%), and a collaborative game style was most preferred by the students (182/497, 36.6%). Three-dimensional game perspectives (270/497, 54.3%) were more popular than two-dimensional perspectives (221/497, 44.5%), especially among males than females (126/185, 68.1% vs 142/303, 46.9%, *P*<.001). In terms of choice for a pharmacy-related serious game, a post-apocalyptic fantasy game (scenario B, 287/497, 57.7%) was more popular than an authentic simulation game (scenario A, 209/497, 42.1%). More males preferred the post-apocalyptic fantasy scenario than females (129/187, 69.0% vs 155/306, 50.7%, *P*<.001).

**Conclusions:**

In general, students want a three-dimensional, fantasy/medieval/mythic post-apocalyptic game, based on an adventurer storyline with an unlocking mechanism reward system. A balance between real-life and fantasy elements needs to be struck in order for the game to cater students towards health care practices.

## Introduction

 As we embrace the digital age, the use of serious games has become increasingly popular in many domains, including education, health care, defense, art and culture, religion, corporate training, and advertising [1,2]. Serious games are digital games that have a purpose beyond entertaining the player [1] such as teaching, training, promoting health and well-being, promoting change, and persuading [3]. Serious games can potentially serve as powerful tools in education because they are motivating and can provide a safe environment for students to learn from their mistakes without having to experience any negative consequences from their actions [4]. Serious games can also be adapted to different learning styles of learners. By matching the learning environment to the ways in which students process and engage with educational material, serious games can provide a learner-centered approach that can supplement didactic teaching methods [5-7].

There are many examples of how serious games have been used for health care education. By creating different clinical reasoning pathways using virtual environments, serious games have potential to train clinical reasoning skills [8]. For example, serious games have been used to train medical students in pediatric medicine [9], insulin management [10], and surgical skills such as coronary artery bypasses and knee arthroplasties [11,12]. Nursing students have used simulation games for training on life support procedures [13] and emergency medical services [14]. Both medical and nursing students have shown to be supportive of the use of serious games in their education [14-16]. In pharmacy education, physical quiz-based games, card games, and board games have been used to teach metabolic pathways, pharmacotherapeutics, and pharmacokinetics [17-19]. However, to our knowledge, evidence on the use of serious games for pharmacy education is scarce even though pharmacy students have indicated their desire for serious games to be incorporated in their curriculum [20,21]. Although medicine, nursing, and pharmacy are all health care disciplines, existing serious games for medical and nursing students cannot be adopted by pharmacy students because the intricacies of drug compounding and preparation of extemporanous (non-commercially available) products, drug dispensing, medications management, medication labeling and review, and other crucial elements of pharmacy education are not present in serious games for medical and nursing students.

Pharmacy education is still largely instructor-centered and based on didactic, knowledge-based teaching [22-24]. At the National University of Singapore, the only provider of pharmacy education in the country [25], pharmacy undergraduates undergo a 4-year program where content and skills are taught through lectures, tutorials, and practical laboratory sessions. As part of a new theme-based curriculum which includes experiential learning [25], the Department of Pharmacy intends to develop and incorporate a serious game into the pharmacy practice modules for the incoming batch of undergraduates. From our reviews of published literature, there have been no studies that characterized the gaming preferences of pharmacy students on a pharmacy-related serious game. In order for us to develop such a game for pharmacy education, we needed to determine what aspects of gameplay (eg, settings, storylines, perspectives, and styles) would be interesting and motivating for our students to play as part of their modules. This study aimed to determine the preferences of students on the gaming aspects and type of pharmacy-related serious game they would like to play as part of their pharmacy practice education. The data collected would then serve as guidance for the development of a pharmacy-related serious game catering to the preferences of pharmacy undergraduates at our institution.

## Methods

### Questionnaire Design

The questionnaire consisted of 13 questions split into two main sections. The first obtained demographic information about the participants and whether they had paid for any in-game items before. If they had, they were asked to indicate for which games and how they paid for them (eg, parents’ money, own allowance, own income). Their interest in playing a pharmacy-related serious game was also obtained using a 5-point Likert scale indicating their interest (1 being not interested at all and 5 being extremely interested).

The second section obtained the preferences of participants regarding various aspects of gameplay for a pharmacy-related serious game, such as their preferred game reward systems, settings, storylines, perspectives, and styles (Textbox 1). These aspects were adapted from classifications defined in the literature [26,27] and modified based on input from the game design team. In addition, participants were asked to choose between two hypothetical scenarios conceived for the serious game: scenario A (authentic simulation) and scenario B (post-apocalyptic fantasy). Scenario A was designed in accordance with the principles of the authentic learning environment theory [28-30], whereas scenario B was a fantasy scenario designed for students who prefer a nonauthentic learning environment. The themes for scenario B were inspired based on the popularity of post-apocalypse and vampirism tropes in the media at the time of the study [31-33]. The survey questions were designed by the study team, and the survey was pre-tested on a small group of science students who were not majoring in the pharmacy course.

**Descriptions of the gameplay aspects obtained from survey respondents.**

Reward system—how the game provides positive experiences to the player

- Score: number used to mark quality of player performance
- Experience points: points accumulated during gameplay that reflect effort and time invested into the game. Usually used to mark the growth and development of the player.
- Item granting: the acquirement of virtual items that can be used by players. These items can possess useful in-game properties and abilities, collectability value, and/or social comparison value.
- Resources: valuables that are collected primarily for practical game use
- Achievement system: titles that are given to players upon completing certain stated conditions
- Feedback messages: fleeting pictures, sounds, or animations during gameplay that evoke positive emotions
- Plot animations and pictures: visually attractive animations and pictures that serve as milestones for player achievement. These usually follow important events in the game.
- Unlocking mechanism: access to new game content such as new levels and minigames awarded when certain conditions are fulfilled. The content motivates the player by evoking and maintaining curiosity towards the game.

Game setting—environment and background in which the player is immersed

- Science fiction: based on futuristic technology, space and time travel, extraterrestrial life, etc.
- Historical: based on real historical persons or events
- Fantasy/medieval/mythic: based on magic and other supernatural phenomena. Includes magical/mythical creatures such as giants, elves, and dragons.
- Modern: setting similar to an authentic, present-day pharmacy

Game storyline—plot and overarching theme in the game

- War: player is a warrior in a violent, organized conflict involving two or more factions.
- Heroic/saving humanity: player must defeat a great evil to save the world.
- Spy/secret agent: player must complete missions while remaining covert and discreet.
- Adventurer: player explores a largely unknown world.
- Authentic pharmacy-related plot: player carries out a role in a workplace that is closely related to an actual pharmacy.

Game perspective—planes along which gameplay action occurs

- 2D game perspectives: gameplay action occurs along a 2D plane only.
  - 2D top-down: camera angle displays the player’s avatar and the surrounding area from above.
  - 2D side-scrolling: camera angle is from the side of the player’s avatar.
- 3D game perspectives: gameplay action occurs in a 3D axis.
  - 3D first-person: camera angle shows the perspective from the viewpoint of the player’s avatar.
  - 3D third-person: camera angle depicts a view that is slightly behind and above the player’s avatar.

Game style—interactivity of how the game is played

- Competitive: players form strategies and directly oppose other players in the game.
- Cooperative: players work together but the benefits from collective efforts are not necessarily shared equally.
- Collaborative: players work together while sharing all payoffs and outcomes.

Game scenario—hypothetical scenarios for a pharmacy-related serious game

- Scenario A (authentic simulation): set in an authentic, modern day pharmacy workplace with a dramatic plot. The goal of the game is to experience the day-to-day operations of a pharmacy. Students will manage contemporary, realistic social issues such as drug addiction, haze, and epidemics. In-game tasks will include activities involving compounding, communication, and pharmaceutical care management.
- Scenario B (post-apocalyptic fantasy): set in a post-apocalyptic 3050, where a pandemic has turned the majority of humans into bloodthirsty vampires. To survive, the remaining humans have learned to use herbs to produce synthetic blood to satisfy the vampires’ craving for human blood. The goal of the game is to find a remedy to reverse the vampiric mutation and to save mankind. In-game tasks will include activities involving compounding, communication, and pharmaceutical care management.

### Study Design

This was a cross-sectional census study using a self-administered survey. Pharmacy students from each of the 4 years of undergraduate study were recruited for the study. Emails were sent to the lecturers-in-charge to seek support and permission to conduct the survey post-lecture. A short briefing would be given regarding the background and aims of the study before distributing the questionnaires. Participation was voluntary and submission of the questionnaire would be considered as consent to the survey. Ethics approval was obtained from the university’s Institutional Review Board.

### Data Analysis

Students were divided into groups based on their demographic information and responses to the survey questions. Their year of study was categorized into lower (years 1 and 2) and upper batches (years 3 and 4). Their interest levels in playing a pharmacy-related serious game were categorized as those who were not interested (not interested) or interested (slightly interested, moderately interested, very interested, and extremely interested). Among those who were interested, interest levels were further split into 2 categories: weak interest (slightly interested and moderately interested) and strong interest (very interested and extremely interested). Students were also classified into two groups by their preferred game perspectives: two-dimensional (2D) top-down or side-scrolling and three-dimensional (3D) first-person or third-person perspectives. Students who chose scenarios A and B were known as scenario A students and scenario B students, respectively.

Results were analyzed using descriptive statistics. Chi-squared tests were used to determine the associations between preferred gameplay aspects and gender (males and females), year of study (lower and upper batches), and preferred game scenarios (scenario A and scenario B students). Statistical significance was defined as **P*<.*05. As the questions on gaming preferences were single-choice questions, multiple responses were excluded from analyses. Similarly, missing responses were also excluded from analyses. All analyses were conducted using the Statistical Product and Service Solutions Version 21 (IBM) software.

## Results

### Demographics and Preferred Gaming Aspects of Respondents

Response rate was 72.7% (497/684 students). More than half were females (307/497, 61.8%) (Table 1). There were almost equivalent proportions of lower batch (249/497, 50.1%) and upper batch (248/497, 49.9%) students. The majority (450/490, 90.5%) was interested in playing a pharmacy-related serious game, among which 65.1% indicated weak interest and 34.9% indicated strong interest. Over one-fifth (118/497, 23.7%) of students had paid for in-game items before, among which most had used their own allowance (101/118, 85.6%) to pay for the items.

The top 2 most popular reward systems were unlocking mechanisms (112/497, 22.5%) and experience points (90/497, 18.1%). Fantasy/medieval/mythic game settings were the most popular (253/497, 50.9%), followed by modern settings (117/497, 23.5%). Almost one-third (147/497, 29.6%) of respondents preferred an adventurer storyline over an authentic pharmacy-related plot (119/497, 23.9%). Three-dimensional game perspectives were more popular than 2D game perspectives (270/497, 54.3% vs 221/497, 44.5%), within which 3D first-person (147/270, 54.4%) and 2D top-down (158/221, 71.5%) views were the more popular ones. A collaborative game style was the most popular (182/497, 36.6%), and scenario B (post-apocalyptic fantasy) was preferred over scenario A (authentic simulation) (287/497, 57.7% vs 209/497, 42.1%).

**Table 1:** Demographics and preferred gaming aspects of respondents.

Parameters			Total^a^, N=497, n (%)
**Gender**			
	Male		187 (37.6)
	Female		307 (61.8)
**Year of study**			
	Year 1		134 (27.0)
	Year 2		115 (23.1)
	Year 3		126 (25.4)
	Year 4		122 (24.5)
**Interest in playing**			
	Not interested		40 (8.0)
	**Interested**		450 (90.5)
		Weak interest (n=450)	293 (65.1)
		Strong interest (n=450)	157 (34.9)
**Paid for in-game** **items**			
	No		379 (76.3)
	**Yes**		118 (23.7)
		Own allowance (n=118)	101 (85.6)
		Own income (n=118)	20 (16.9)
		Parents’ money (n=118)	13 (11.0)
**Reward system**			
	Score		65 (13.1)
	Experience points		90 (18.1)
	Item granting		42 (8.5)
	Resources		23 (4.6)
	Achievement system		60 (12.1)
	Feedback messages		5 (1.0)
	Plot animations & pictures		38 (7.6)
	Unlocking mechanism		112 (22.5)
**Game setting**			
	Science fiction		67 (13.5)
	Historical		39 (7.8)
	Fantasy/medieval/mythic		253 (50.9)
	Modern		117 (23.5)
	Others		2 (0.4)
**Game storyline**			
	War		50 (10.1)
	Heroic/saving humanity		61 (12.3)
	Spy/secret agent		99 (19.9)
	Adventurer		147 (29.6)
	Authentic pharmacy-related plot		119 (23.9)
	Others		5 (1.0)
**Game perspective**			
	**2D**		221 (44.5)
		2D top-down, (n=221)	158 (71.5)
		2D side-scrolling (n=221)	63 (28.5)
	**3D**		270 (54.3)
		3D first-person (n=270)	147 (54.4)
		3D third-person (n=270)	123 (45.6)
**Game style**			
	Competitive		147 (29.6)
	Cooperative		160 (32.2)
	Collaborative		182 (36.6)
**Game scenario**			
	A (authentic simulation)		209 (42.1)
	B (post-apocalyptic fantasy)		287 (57.7)

^a^ Percentages may not add to 100% due to missing data or multiple responses.

###  Analysis by Gender

Among respondents who were interested in playing a pharmacy-related game, males had a stronger interest than females (77/169, 45.6% vs 79/278, 28.4%, **P*<.*001) (Table 2). Males were also more likely to have paid for in-game items compared to females (59/187, 31.6% vs 59/307, 19.2%, **P*=.*002), and they were more likely to have used their own income to pay for these items (14/58, 24.1% vs 6/59, 10.2%, **P*=.*045).

There was a trend in preference for reward systems. Males preferred experience points (31/157, 19.7%) to unlocking mechanisms (29/157, 18.5%); the opposite was true for females, who preferred unlocking mechanisms (83/275, 30.2% vs 59/275, 21.5%). While males were less likely to want unlocking mechanisms for the game (29/159, 18.5% vs 83/275, 30.2%, **P*=.*008), they were more likely to want rewards of item granting (22/157, 14.0% vs 20/275, 7.3%, **P*=.*023) and plot animations and pictures (19/157, 12.1% vs 18/275, 6.5%, **P*=.*047).

A fantasy/medieval/mythic game setting with an adventurer storyline was the most popular in both genders, followed by a modern setting with an authentic pharmacy-related plot (Table 2). In addition, males preferred storylines with a war component more (38/183, 20.8% vs 11/295, 3.7%, **P*<.*001) and spy/secret agent settings less (23/183, 12.6% vs 76/295, 25.8%, **P*<.*001).

The perspectives and game styles were similar for both genders, with 2D top-down and 3D first-person views and a collaborative style being the most popular. However, in terms of game perspectives, 3D views were more popular among males than females (126/185, 68.1% vs 142/303, 46.9%, **P*<.*001). Similarly, while both genders preferred scenario B (post-apocalyptic fantasy) for the pharmacy-related game, males were more likely choose this scenario than females (129/187, 69.0% vs 155/306, 50.7%, **P*<.*001).

**Table 2:** Comparison of gaming aspects by gender.

Gaming aspects		Male (N=187), n (%)	Female (N=307), n (%)	*P* values
**Interest in** **playing**				
	Not interested	17/186 (9.1)	23/301 (7.6)	.558
	Interested	169/186 (90.9)	278/301 (92.4)	
**Interest level** **of interested** **students**				
	Weak interest	92/169 (54.4)	199/278 (71.6)	<.001^b^
	Strong interest	77/169 (45.6)	79/278 (28.4)	
**Paid for** **in-game items**				
	Yes	59/187 (31.6)	59/307 (19.2)	.002^b^
	No	128/187 (68.4)	248/307 (80.8)	
**Payment** **method for** **in-game items** ^a^				
	Own allowance	50/58 (86.2)	51/59 (86.4)	.971
	Own income	14/58 (24.1)	6/59 (10.2)	.045^b^
	Parents’ money	6/58 (10.3)	7/59 (11.9)	.794
**Reward** **system**				
	Score	26/157 (16.6)	39/275 (14.2)	.506
	Experience points	31/157 (19.7)	59/275 (21.5)	.674
	Item granting	22/157 (14.0)	20/275 (7.3)	.023^b^
	Resources	7/157 (4.5)	15/275 (5.5)	.651
	Achievement system	23/157 (14.6)	37/275 (13.5)	.730
	Feedback messages	0 (0.0)	4/275 (1.5)	.302
	Plot animations & pictures	19/157 (12.1)	18/275 (6.5)	.047^b^
	Unlocking mechanism	29/157 (18.5)	83/275 (30.2)	.008^b^
**Game setting**				
	Science fiction	30/182 (16.5)	36/293 (12.3)	.199
	Historical	18/182 (9.9)	21/293 (7.2)	.293
	Fantasy/medieval/mythic	98/182 (53.8)	153/293 (52.2)	.730
	Modern	36/182 (19.8)	81/293 (27.6)	.053
	Others	0 (0.0)	2/293 (0.7)	-
**Game storyline**				
	War	38/183 (20.8)	11/295 (3.7)	<.001^b^
	Heroic/saving humanity	25/183 (13.7)	35/295 (11.9)	.564
	Spy/secret agent	23/183 (12.6)	76/295 (25.8)	<.001^b^
	Adventurer	54/183 (29.5)	93/295 (31.5)	.642
	Authentic pharmacy-related plot	41/183 (22.4)	77/295 (26.1)	.362
	Others	2/183 (1.1)	3/295 (1.0)	-
**Game** **perspective**				
	2D	59/185 (31.9)	161/303 (53.1)	<.001^b^
	3D	126/185 (68.1)	142/303 (46.9)	
**2D game** **perspectives**				
	2D top-down	48/59 (81.4)	110/161 (68.3)	.057
	2D side-scrolling	11/59 (18.6)	51/161 (31.7)	
**3D game perspectives**				
	3D first-person	67/126 (53.2)	79/142 (55.6)	.687
	3D third-person	59/126 (46.8)	63/142 (44.4)	
**Game style**				
	Competitive	54/182 (29.7)	93/304 (30.6)	.830
	Cooperative	56/182 (30.8)	101/304 (33.2)	.575
	Collaborative	72/182 (39.6)	110/304 (36.2)	.457
**Game scenario**				
	A (authentic simulation)	58/187 (31.0)	151/306 (49.3)	<.001^b^
	B (post-apocalyptic fantasy)	129/187 (69.0)	155/306 (50.7)	

^a^Percentages may not add to 100% due to multiple responses.

^b^Statistical significance was defined as **P*<.*05.

###  Analysis by Year of Study (Lower and Upper Batches)

Lower batch students were more likely to have paid for in-game items compared to upper batch students (70/245, 28.1% vs 48/245, 19.4%, **P*=.*022) (Table 3). However, among those who paid for in-game items, the lower batch was less likely to have used their own income (8/70, 11.4% vs 12/47, 25.5%, **P*=.*047).

An experience points reward system (54/214, 25.2%) was more popular for lower batch students compared to unlocking mechanisms (45/214, 21.0%). In contrast, the experience points system was less popular among upper batch students (36/221, 16.3% vs 67/221, 30.3%). Lower batch students were more likely to want an experience points-based reward (54/214, 25.2% vs 36/221, 16.3%, **P*=.*021) than an unlocking mechanism reward (45/214, 21.0% vs 67/221, 30.3%, **P*=.*027).

Fantasy/medieval/mythic and modern game settings were the most popular among both lower and upper batch students (Table 3). However, lower batch students preferred modern settings less than their upper batch counterparts (47/236, 19.9% vs 70/242, 28.9%, **P*=.*022). The adventurer game storyline was the most popular among lower batch students (75/237, 31.6%), but an authentic pharmacy-related plot was more popular than the adventurer storyline among the upper batch students (77/244, 31.6% vs 72/244, 29.5%). In fact, upper batch students were more likely to prefer the authentic pharmacy-related plot (77/244, 31.6% vs 42/237, 17.7%, **P*<.*001). On the other hand, lower batch students preferred a spy/secret agent storyline more than their seniors (58/237, 24.5% vs 41/244, 16.8%, **P*=.*038). In terms of game scenario preferences, while scenario B (post-apocalyptic fantasy) was generally more popular for both batches, lower batch students were more open to playing the post-apocalyptic fantasy scenario in the pharmacy-related game (161/248, 64.9% vs 126/248, 50.8%, **P*=.*001).

**Table 3:** Comparison of gaming aspects by year of study.

Gaming aspects		Lower batch (N=249), n (%)	Upper batch (N=248), n (%)	*P* values
**Interest in playing**				
	Not interested	22/245 (9.0)	18/245 (7.3)	.509
	Interested	223/245 (91.0)	227/245 (92.7)	
**Interest level of interested students**				
	Weak interest	139/223 (62.3)	154/227 (67.8)	.220
	Strong interest	84/223 (37.7)	73/227 (32.2)	
**Paid for in-game items**				
	Yes	70/245 (28.1)	48/245 (19.4)	.022^b^
	No	179/245 (71.9)	200/245 (80.6)	
**Payment method for in-game items** ^a^				
	Own allowance	59/70 (84.3)	42/47 (89.4)	.433
	Own income	8/70 (11.4)	12/47 (25.5)	.047^b^
	Parents’ money	9/70 (12.9)	4/47 (8.5)	.463
**Reward system**				
	Score	31/214 (14.5)	34/221 (15.4)	.793
	Experience points	54/214 (25.2)	36/221 (16.3)	.021^b^
	Item granting	24/214 (11.2)	18/221 (8.1)	.278
	Resources	10/214 (4.7)	13/221 (5.9)	.573
	Achievement system	28/214 (13.1)	32/221 (14.5)	.673
	Feedback messages	0 (0.0)	5/221 (2.3)	.061
	Plot animations & pictures	22/214 (10.3)	16/221 (7.2)	.262
	Unlocking mechanism	45/214 (21.0)	67/221 (30.3)	.027^b^
**Game setting**				
	Science fiction	35/236 (14.8)	32/242 (13.2)	.613
	Historical	18/236 (7.6)	21/242 (8.7)	.675
	Fantasy/medieval/mythic	135/236 (57.2)	118/242 (48.8)	.064
	Modern	47/236 (19.9)	70/242 (28.9)	.022^b^
	Others	1/236 (0.4)	1/242 (0.4)	-
**Game storyline**				
	War	24/237 (10.1)	26/244 (10.7)	.849
	Heroic/saving humanity	35/237 (14.8)	26/244 (10.7)	.175
	Spy/secret agent	58/237 (24.5)	41/244 (16.8)	.038^b^
	Adventurer	75/237 (31.6)	72/244 (29.5)	.611
	Authentic pharmacy-related plot	42/237 (17.7)	77/244 (31.6)	<.001^b^
	Others	3/237 (1.3)	2/244 (0.8)	-
**Game perspective**				
	2D	109/245 (44.5)	112/246 (45.5)	.817
	3D	136/245 (55.5)	134/246 (54.5)	
**2D game** **perspectives**				
	2D top-down	75/109 (68.8)	83/112 (74.1)	.383
	2D side-scrolling	34/109 (31.2)	29/112 (25.9)	
**3D game** **perspectives**				
	3D first-person	73/136 (53.7)	74/134 (55.2)	.799
	3D third-person	63/136 (46.3)	60/134 (44.8)	
**Game style**				
	Competitive	71/243 (29.2)	76/246 (30.9)	.686
	Cooperative	84/243 (34.6)	76/246 (30.9)	.387
	Collaborative	88/243 (36.2)	94/246 (38.2)	.648
**Game scenario**				
	A (authentic simulation)	87/248 (35.1)	122/248 (49.2)	.001^b^
	B (post-apocalyptic fantasy)	161/248 (64.9)	126/248 (50.8)	

^a^Percentages may not add to 100% due to multiple responses.

^b^Statistical significance was defined as **P*<.*05.

###  Analysis by Choice of Game Scenario

In general, as the students grew in the number of study years, their choices shifted from scenario B (post-apocalyptic fantasy) to scenario A (authentic simulation) (Table 4). Students in year 1 of the pharmacy course were more likely to want to play a post-apocalyptic fantasy game (90/287, 31.4% vs 43/209, 20.6%, **P*=.*007), while those who were near graduation preferred to play the authentic simulation game (61/209, 29.2% vs 61/287, 21.3%, **P*=.*043).

In terms of rewards, unlocking mechanisms was the most popular choice among scenario A students (57/184, 31.0%), while experience points was the most popular among scenario B students (57/250, 22.8%). Students who chose the authentic simulation scenario (scenario A) were more likely to want an unlocking mechanism reward system for the game compared to those who chose the post-apocalyptic scenario (scenario B) (57/184, 31.0% vs 55/250, 22.0%, **P*=.*035). A modern game setting was preferred by scenario A students (90/201, 44.8% vs 27/276, 9.8%, *P*<.001), in contrast to a fantasy/medieval/mythic setting, which was the more preferable choice among scenario B students (187/276, 67.8% vs 66/201, 32.8%, **P*<.*001).

An authentic pharmacy-related plot was the most popular among scenario A students (86/201, 42.8%) but the least popular among scenario B students (33/279, 11.8%). Scenario A students were more likely to want an authentic pharmacy-related plot for the game (86/201, 42.8% vs 33/279, 11.8%, **P*<.*001), while scenario B students were more likely to want war (38/279, 13.6% vs 12/201, 6.0%, **P*=.*007), heroic/saving humanity (44/279, 15.8% vs 17/201, 8.5%, **P*=.*018), or spy/secret agent (67/279, 24.0% vs 31/201, 15.4%, **P*=.*021) storylines. Those who chose authentic simulations (scenario A) preferred the game to be in a 2D perspective (119/207, 57.5%), compared to those who chose the post-apocalyptic fantasy (scenario B), who preferred it in a 3D perspective (182/283, 64.3%, **P*<.*001). The latter group (scenario B students) were also more likely to want a competitive style (95/281, 33.8% vs 52/207, 25.1%, **P*=.*039) for the game.

**Table 4:** Comparison of gaming aspects by game scenario choice in the pharmacy-related serious game.

Gaming aspects		Scenario A (N=209), n (%)	Scenario B (N=287), n (%)	*P* values
**Year of study**				
	Year 1	43/209 (20.6)	90/287 (31.4)	.007^a^
	Year 2	44/209 (21.1)	71/287 (24.7)	.337
	Year 3	61/209 (29.2)	65/287 (22.6)	.099
	Year 4	61/209 (29.2)	61/287 (21.3)	.043^a^
**Interest in playing**				
	Not interested	20/206 (9.7)	19/283 (6.7)	.227
	Interested	186/206 (90.3)	264/283 (93.3)	
**Interest level of interested students**				
	Weak interest	126/186 (67.7)	167/264 (63.3)	.326
	Strong interest	60/186 (32.3)	97/264 (36.7)	
**Paid for in-game items**				
	Yes	41/209 (19.6)	77/287 (26.8)	.063
	No	168/209 (80.4)	210/287 (73.2)	
**Payment method for in-game items**				
	Own allowance	39/40 (97.5)	62/77 (80.5)	.010^a^
	Own income	6/40 (15.0)	14/77 (18.2)	.665
	Parents’ money	1/40 (2.5)	12/77 (15.6)	.034^a^
**Reward** **system**				
	Score	31/184 (16.8)	34/250 (13.6)	.349
	Experience points	33/184 (17.9)	57/250 (22.8)	.217
	Item granting	13/184 (7.1)	28/250 (11.2)	.146
	Resources	11/184 (6.0)	12/250 (4.8)	.588
	Achievement system	24/184 (13.0)	36/250 (14.4)	.686
	Feedback messages	3/184 (1.6)	2/250 (0.8)	.654
	Plot animations & pics	12/184 (6.5)	26/250 (10.4)	.158
	Unlocking mechanism	57/184 (31.0)	55/250 (22.0)	.035^a^
**Game setting**				
	Science fiction	23/201 (11.4)	43/276 (15.6)	.196
	Historical	22/201 (10.9)	17/276 (6.2)	.060
	Fantasy/medieval mythic	66/201 (32.8)	187/276 (67.8)	<.001^a^
	Modern	90/201 (44.8)	27/276 (9.8)	<.001^a^
	Others	0 (0.0)	2/276 (0.7)	-
**Game storyline**				
	War	12/201 (6.0)	38/279 (13.6)	.007^a^
	Heroic/saving humanity	17/201 (8.5)	44/279 (15.8)	.018^a^
	Spy/secret agent	31/201 (15.4)	67/279 (24.0)	.021^a^
	Adventurer	55/201 (27.4)	92/279 (33.0)	.188
	Authentic pharmacy-related plot	86/201 (42.8)	33/279 (11.8)	<.001^a^
	Others	0 (0.0)	5/279 (1.8)	-
**Game** **perspective**				
	2D	119/207 (57.5)	101/283 (35.7)	<.001^a^
	3D	88/207 (42.5)	182/283 (64.3)	
**2D game perspectives**				
	2D top-down	87/119 (73.1)	71/101 (70.3)	.644
	2D side-scrolling	32/119 (26.9)	30/101 (29.7)	
**3D game perspectives**				
	3D first-person	54/88 (61.4)	93/182 (51.1)	.112
	3D third-person	34/88 (38.6)	89/182 (48.9)	
**Game style**				
	Competitive	52/207 (25.1)	95/281 (33.8)	.039^a^
	Cooperative	68/207 (32.9)	91/281 (32.4)	.914
	Collaborative	87/207 (42.0)	95/281 (33.8)	.063

^a^Statistical significance was defined as **P*<.*05.

### Analysis by Past Payment for In-Game Items

Out of the 497 respondents, 118 (23.7%) had previously paid for in-game items. Males were more likely than females to have paid for in-game items (59/187, 31.6% vs 59/307, 19.2%, **P*=.*002) (Table 2). Lower batch students were also more likely to have paid for in-game items compared to upper batch students (70/245, 28.1% vs 48/245, 19.4%, **P*=.*022) (Table 3). Among the 110 students who indicated the games in which they had made in-game purchases, the most popular game by 57 respondents (51.8%) was MapleStory.

A fantasy/medieval/mythic game setting was preferred by students who had paid for in-game items (80/118, 67.8% vs 186/377, 49.3%, **P*<.*001), in contrast to a modern setting, which was the preferred choice among students who had never paid for in-game items (108/377, 28.6% vs 16/118, 13.6%, **P*=.*001). For game storyline, students with past payment for in-game items preferred war (20/118, 16.9% vs 35/377, 9.3%, **P*=.*021) and adventurer (48/118, 40.7% vs 112/377, 29.7%, **P*=.*026) storylines; authentic storylines were preferred by students who had never paid for in-game items (105/377, 27.9% vs 18/118, 15.3%, **P*=.*006).

## Discussion

### Developing a Pharmacy-Related Serious Game Based on Gaming Preferences of Students

The gaming behaviors and preferences of students have been extensively documented in the literature [15,34-40]. However, these studies have predominantly focused on recreational, not serious, gaming. The focus of serious games is on a purpose other than entertainment, in this case pharmacy education. Although certain recreational gaming preferences can also help explain students’ gaming preferences for a serious game, different categorizations of gaming aspects can render direct comparisons between the two types of gameplay difficult. For example, role-playing recreational games can be further split into fantasy/medieval/mythic (eg, World of Warcraft) or science fiction (eg, Eve Online) settings [15,41,42]. In order for us to develop a serious game for pharmacy education, we needed to find out more about the types of gameplay aspects (eg, settings, storylines, perspectives, and styles) that would be relevant. Based on our literature reviews, we could not find any study results that could be directly translated for us to develop such a game. Studies on gaming in medical and nursing education were generally focused on simulations and not role-playing. Furthermore, the gaming preferences of students in different health care fields might differ. To our knowledge, this is the first study that has identified the preferences of students in relation to gaming aspects and the associations between gaming aspects and gender, year of study, and choice of hypothetical game scenarios. In addition, this is the first survey conducted on an Asian population with regards to serious games, and it may help lay the foundation for more studies. In general, the most popular game reward systems and game settings were unlocking mechanisms and fantasy/medieval/mythic settings. Students generally preferred to play an adventurer game in a collaborative mode and from a 3D viewing perspective.

Our results suggested that recreational gaming behavior might also explain serious gaming preferences. The most popular game among students who had paid for in-game items was MapleStory, which has a fantasy/medieval/mythic setting and an adventurer storyline [43,44]. For the pharmacy-related serious game, these gaming aspects were also preferred by these students compared to their counterparts who had not previously paid for in-game items.

Generally, both genders shared the same gaming preferences. The difference in gaming perspectives between males and females, who preferred 3D and 2D perspectives respectively, could be due to the types of gaming platforms used by both genders for recreational gaming. Studies have shown that males were more likely to play games on advanced gaming equipment, such as the Microsoft Xbox and PlayStation gaming consoles [15,34,35]. The recreational gaming preferences of the students could also have played a role in their perceptions of secondary gaming aspects. For example, the higher preference for war storylines by males could be linked to the fact that they played violent video games for recreation more frequently [36]. The preference of males towards choosing a post-apocalyptic fantasy (scenario B) could also be related to their preference for games that involved fantasy genres [40]. On the other hand, the preference of females for an authentic simulation (scenario A) game in pharmacy could mimic the fact that they liked games which involved close simulation of real-life activities [45].

While both the lower and upper batches of students showed similarities in preferences for the gaming aspects, their preferences for the game storyline and setting were quite different. The lower and upper batch students chose the adventurer storyline and authentic pharmacy-related plot respectively, and the upper batch also preferred the intended game to portray a modern setting similar to a present-day pharmacy. Their preferences could be related to the fact that younger players tended to be more motivated by fantasy elements and required more stimulation than older players when playing video games [37,46]. However, the shift in choice of scenario B (post-apocalyptic fantasy) to scenario A (authentic simulation) for the pharmacy game among the student batches showed their maturity in attitude as they neared graduation. Their preferences for fantasy elements decreased, and they were more attracted to realistic and authentic elements as they neared the transit from undergraduate study to working life.

The differences in preferences among the genders and batches make it a challenge to design a serious game that caters to all pharmacy students. Our results show diversity in gaming preferences, but there are several aspects that can be generalized across the groups. The popularity of realistic and authentic gaming aspects suggests that the final version of the serious game should incorporate elements that mirror that of real-life pharmacy practices. The differences in preferences on reward systems may imply that it is necessary to incorporate an array of reward systems in the final game. In fact, the use of multiple reward systems already exists for current recreational games in the market. For example, Diablo 3 (Blizzard Entertainment) primarily uses an item-granting reward system, where players obtain rare and gameplay-enhancing items by defeating enemies. At the same time, the players can level up their characters by gaining experience points, compete against other players in a leaderboard scoring system, and collect game rewards through achievement and completion of game events. Nevertheless, it will still be a challenge to design a game that caters towards the variety of preferences. While some gaming aspects can be tailored to the player (ie, 3D and 2D perspectives for males and females), other aspects will need to change as the game evolves and caters to a more mature audience (ie, from a fantasy to a more authentic storyline and setting). For our pharmacy game, it will be important to incorporate a variety of reward systems and a mixture of fantasy and authentic elements in order to encourage students to achieve the learning objectives of the module.

### Limitations

A major limitation in this study is that our results are mainly focused on the gaming aspects that students prefer for a serious game. Taking into consideration that the serious game is intended for educational purposes, other characteristics such as the learning styles of students can affect their gaming preferences. For example, in the Keirsey temperament sorter personality test [6], students who are Artisans value freedom and spontaneity and therefore may prefer gaming aspects that offer such qualities, such as adventurer storylines. On the other hand, students who are Guardians are traditional and conservative and may prefer gaming aspects that are more grounded in real-life environments, such as having modern settings. However, obtaining such information on our students’ learning styles was not feasible in this study due to the limited time in lectures allocated for the survey. Background information such as duration of gaming experience, recreational gaming habits, and technology aversion was not collected, as we were mindful that the increase in the number of questions might predispose the students to survey fatigue and affect the response rate. Obtaining this information is necessary, however, and will be a next step for us in order to cater the game successfully towards its educational purpose.

Another limitation is that our results may have limited generalizability to pharmacy students in other countries. However, our university houses the only pharmacy school in the country, and our department produces majority of the country’s pharmacy workforce. Therefore, the results of this study are still applicable to the cohorts of students entering pharmacy school in Singapore. Our survey is also valuable as a basic framework that can be used to obtain the perceptions of serious games of other populations of pharmacy students. Future work can obtain quantitative information regarding students’ recreational gaming experiences and habits so that more detailed analyses, such as regression models, can be performed to obtain further insights.

### Conclusion

This is the first study that has attempted to characterize the preferences of pharmacy students on various gaming aspects for a serious game in their education. In general, students want a game with the following combination of gaming aspects: an unlocking mechanism reward system, a fantasy/medieval/mythic setting, an adventurer storyline, a 3D viewing perspective, and a collaborative game style. Students prefer a post-apocalyptic fantasy scenario over an authentic simulation scenario, although this preference shifts as the students mature over the course of their undergraduate years. A balance between real-life environments and fantasy aspects will need to be struck.

## Abbreviations

2D: two-dimensional
3D: three-dimensional
